# Effect of ammoniation and harvest method on waste and consumption of corn residue bales fed to cows in a round bale feeder

**DOI:** 10.1093/tas/txaa047

**Published:** 2020-04-18

**Authors:** Ashley C Conway, Zac Carlson, Fred Hilscher, Jim C MacDonald, Terry J Klopfenstein, Mary E Drewnoski

**Affiliations:** Department of Animal Science, University of Nebraska-Lincoln, Lincoln, NE

**Keywords:** ammoniation, beef cow, corn residue, intake

## Abstract

To determine the effects of harvest method and ammoniation (3.7% of dry matter) on consumption and waste of baled corn residue, a 6 × 6 Latin square with a 3 × 2 factorial treatment structure was conducted. Six treatments consisted of either nonammoniated or ammoniated residue, harvested one of three ways: conventional rake and bale (CONV), New Holland Cornrower with two rows of stem chopped into the windrow with tailings (2ROW), or EZBale system (EZB) with a disengaged combine spreader and tailings dropped in a windrow. Open cows were grouped by body weight to produce a light block of two pens (448 kg ± 49.6) and a heavy block of four pens (649 kg ± 65.9). One bale was fed to each pen during each of six 7-d periods using round bale ring feeders with closed bottom panels. Residue falling around (waste) and remaining in (refusals) the feeder was collected. The daily nutrient intake was estimated as the difference between what was offered and what remained (waste plus refusals). Crude protein (CP) of residue offered did not differ (*P* = 0.58) among harvest methods. The digestible organic matter (DOM) content of residue offered in 2ROW and EZB bales did not differ (*P* = 0.86) and was greater (*P* < 0.01) than CONV. Ammoniation increased (*P* < 0.01) CP and DOM content of the residue offered. Total wasted and refused residue did not differ (*P* = 0.12) between 2ROW (29%) and EZB (37%), while CONV (42%) was greater (*P* = 0.02) than 2ROW but did not differ (*P* = 0.34) from EZB. Ammoniation reduced (*P* = 0.03) total waste and refusals from 41% to 32%. The nutrient content of both waste and refusals did not differ (*P* ≥ 0.34) among harvest methods and, with the exception of CP, was not affected (*P* ≥ 0.15) by ammoniation. The CP content of the waste was greater (*P* = 0.02) and refusals tended to be greater (*P* = 0.08) from ammoniated bales. The CP intake of 2ROW was greater (*P* ≤ 0.02) than both EZB and CONV, while EZB tended (*P* = 0.06) to be greater than CONV. The CP intake of all ammoniated residues was greater (*P* < 0.01) than the nonammoniated residue. The DOM intake of nonammoniated 2ROW and EZB did not differ (*P* = 0.61) but was greater than nonammoniated CONV (*P* < 0.01). Ammoniation increased (*P* < 0.01) DOM intake. Overall, ammoniation had much larger effects than harvest method, resulting in reduced waste and refusals and greater intake of DOM and CP. However, the combination of both ammoniation and selective harvest (2ROW or EZB) was needed to result in energy and protein intakes that would meet the needs of a mature cow in mid-gestation.

## INTRODUCTION

Feed costs are the most critical control point for profitability in beef cattle production, and costs associated with winter feeding can be particularly high (May et al., 1999; Ramesy et al., 2005). These costs can be reduced by fall or winter corn residue grazing, which is currently the most economical option for corn residue utilization (Schmer et al., 2017; Redfearn et al., 2019). Alternatively, baled corn residue can offer low-cost forage to cattle producers who may not have access to grazing acres. Previous work has evaluated baled corn residues when fed after grinding and mixing into a total mixed ration (King et al., 2017; Conway et al., 2019). Little information is available on the feeding value and waste of whole bales of corn residue fed in ring feeders, which may be more feasible for cattle producers who cannot graze corn residue and who do not have access to grinding and ration-mixing equipment.

Inherent differences in the nutritive value of the different corn plant parts have been previously noted, with husk being the most digestible, leaf being intermediate, stem being the least digestible, and cob being highly variable (Fernandez-Rivera and Klopfenstein, 1989b; Gutierrez-Ornelas and Klopfenstein, 1991). Selective harvest methods can change the plant part proportion in the corn residue bales, changing the digestibility of the baled corn residue (King et al., 2017; Conway et al., 2019). Furthermore, ammoniation has also been shown to increase intake, digestibility, and crude protein (CP) content of low-quality forages (Saenger et al, 1982; Fahmy and Klopfenstein, 1994) and there is some evidence that it will differentially affect individual corn plant parts (Ramirez et al., 2007; Conway et al., 2019). The objective of this study was to quantify the effects of three different harvest methods and ammoniation on the intake and waste of corn residue when fed to dry cows as a whole bale in a ring feeder.

## MATERIALS AND METHODS

Animal care and management procedures used were reviewed and approved by the University of Nebraska Institutional Care and Animal Use Committee (protocol 1282).

### Corn Residue Harvesting and Ammoniation

Corn residue used in this trial was harvested in October 2016. Residue was baled and removed from two adjacent, nonirrigated fields within 48 h of corn harvest. A total of 40.9 ha of the same corn hybrid were harvested using three different harvest methods. Using a conventional John Deere S550 with a 608 eight-row corn head (John Deere, Moline, IL) followed by a VR1428 High Capacity wheel rake (Vermeer Freeman Manufacturing, Inc., Freeman, SD), 7.3 ha of corn residue were harvested using a conventional rake-and-bale method (CONV). Assuming a harvest index of 0.55, the CONV removed an estimated 77% of the available residue from the field. Another 15.4 ha were harvested using the same John Deere S550 combine with a 608 eight-row corn head (John Deere, Moline, IL) but without the raking for residue removal in a method promoted as the “EZ Bale system” (Poet-DSM Advanced Biofuels, Sioux Falls, SD). This harvest method entails harvesting as normal but disengaging the rear spreader of the combine to drop the tailings and stem and leaf into a windrow and can be followed immediately with a baler. This material was removed at a rate of approximately 34% of available residue, again assuming a harvest index of 0.55, and produced the EZB treatment bales.

Finally, the New Holland Cornrower Corn Head (Straeter, 2011; Craig Welding, Mentone, IN) was used to harvest 18.2 ha. The Cornrower attachment has individual chopping units underneath the corn head, which can be turned on or off in pairs, and the corn stem and leaf that is harvested is chopped and dropped into the resulting windrow. Two rows of stem and leaf were chopped and added to the windrow in this harvest method to produce the 2ROW bales and resulted in approximately 28% residue removal, assuming a harvest index of 0.55.

After baling, 65 bales (19 2ROW, 25 CONV, and 21 EZB) with an average 83% dry matter (DM) were separated and stacked on a concrete pad lined with black plastic. Bales were stacked in a 4 × 3 pyramid arrangement with harvest methods randomly placed in the stack. The stack was covered with the plastic and the edges sealed. Anhydrous ammonia was pumped into the stack at 3.7% of DM and the stack remained sealed for 60 d (12 November 2016 to 11 January 2017).

Previous research has reported that the nutrient composition of the bales differ among harvest methods and is likely due to differences in the amounts of various plant parts. Therefore, samples of approximately 2.5 kg of material from 12 bales (*n* = 4 for each harvest method) were collected to assess the proportions of each plant part in the bales. Total samples were weighed and residue was hand separated into husk, leaf (with sheath), stem, and cob. Residual chaff at the bottom of each sample bag was separated through a 1-mm wire mesh screen. The residue not passing through the screen was considered leaf, and the remaining chaff was weighed. Each plant part was weighed, and subsamples from each part were collected and dried in a 60 °C forced-air oven to determine DM and calculate the proportion of each plant part (on DM basis) in the bales.

### Feeding Trial

A 52-d feeding trial was conducted at the University of Nebraska-Lincoln Eastern Nebraska Research and Extension feedlot facilities near Mead, NE, between August and October of 2017. A total of 42 open commercial cross-bred beef females were used and ranged in age and parity from first-calf heifers to multiparous 7-yr-old cows. The animals came from a single herd owned by the University. Cows had weaned a calf in the fall of the previous year but were being transitioned from spring to fall calving and, thus, were open during the trial. The pool included 12 heifers and 30 cows; the animals were stratified by body weight (BW) and blocked to produce two light blocks (448 kg ± 49.6; 6 heifers and 1 cow per pen) and four heavy blocks (649 kg ± 65.9; 7 cows per pen). Body condition score was 4.5 (SD ± 0.52) when assessed on a 1–9 scale. This resulted in six pens of seven animals. Each pen of animals was allotted two 9.8- × 28-m open-air pens during the feeding trial, which were separated by a combination of electric and fixed fence and gate. Animals alternated pens at the end of each period and were moved to the neighboring pen with their respective feeder in order to assist with pen cleaning and final period sample collection. Each pen had a 9.8- × 6.7-m concrete apron extending from the bunk, and the back of the pen was packed soil.

The experiment was designed as a 6 × 6 Latin square with a 3 × 2 factorial treatment structure with six 1-wk periods. The six treatments were whole round bales of nonammoniated corn residue from one of three different harvest methods (CONV, 2ROW, and EZB) or the ammoniated bales of the same three harvest methods. At the start of the trial, animals were fed whole round bales of conventionally harvested corn residue for 10 d to adapt to the pen conditions and eating bales from the ring feeders. Each pen was supplemented with a commercial mineral–vitamin supplement in the form of a cooked molasses lick tub (average consumption 1.46 kg per cow per day). The supplement contained no added urea or salt (guaranteed analysis: 7.5% CP, 3.0% crude fat, 2.0% crude fiber, 5.0–6.0% Ca, 6.0% P, 1.5% Mg, 4.0% K, 2,100 mg/kg Zn, 1,165 mg/kg Mn, 730 mg/kg Cu, 75 mg/kg Co, 68 mg/kg I, 13 mg/kg Se, 176,320 IU/kg vitamin A, 44,080 IU/kg vitamin D, and 220 IU/kg vitamin E).

At the beginning of each period, every pen received their respective treatment as one whole, unground round bale in a ring feeder with the mesh wrapping removed. All feeders were round bale ring feeders with straight sides and a bottom panel. The feeder was situated in the middle of the concrete apron. Once during the trial, a pen had minimal residue left inside the feeder on the day before the end of the 1-wk period and cows were removed from the pen and offered wheat straw.

### Collection Period Methods and Sampling

Prior to the start of each period, each bale was weighed and core sampled using a 60- × 1.5-cm drill-powered probe (Hay Probe, Hart Machine Company, Madras, OR). Three times during each period, the corn residue falling outside of the feeder was raked and collected (Wednesday, Friday, and Monday). Using household yard leaf rakes, the residue collected during the period was separated visually into “clean” and “contaminated” waste. Clean waste was dry and unsoiled and was put in the feed bunk located at the front of the pens. The “contaminated” waste that was soiled with feces and urine was shoveled to the edge of the pen. At the end of the period, cattle were moved to their alternate pen with their feeder and given their next treatment bale. At this time, the remaining residue waste was collected, and the total weights of the clean and contaminated waste were taken. Any refusals (orts) remaining inside of the ring feeder were also collected and weighed. A subsample of approximately 0.1 m^3^ of material (brown paper grocery bags measuring 26 × 36 × 15 cm) of the clean waste, contaminated waste, and refusals in each pen was collected using the four-corners sampling method. Total residue waste and refusals were adjusted for DM (100 °C) and reported as a percentage of the initial bale weight. Wasted and refused residue values were added together, and this value was subtracted from the total offered DM to estimate residue disappearance as a measurement of animal intake.

### Quality Sample Analysis

Quality samples for clean, contaminated, and refused residue, as well as the bale core samples from each period, were analyzed for DM using a forced-air oven at 60 °C for 48–72 h, with samples being weighed back when there was less than 0.02-g fluctuation between three consecutive weights taken. These samples were then ground through a 1-mm screen using a Wiley mill. True DM was assessed with 24 h in 100 °C oven, and the organic matter (OM) of the samples was measured by incinerating in a 600 °C muffle furnace for 6 h. Neutral detergent fiber (NDF) and acid detergent fiber (ADF) were analyzed using an automated ANKOM 2000 fiber analyzer (ANKOM Technology, Macedon NY). Approximately 0.5000–0.5040 g of each sample was measured in a 25-micron porosity fiber bags and bags were analyzed sequentially with sodium sulfite included in the NDF analysis. Nitrogen content was measured with an N/protein configured FlashSmart elemental analyzer (Thermo Fischer Scientific, Inc.) using dynamic flash combustion (Dumas method) with Ethylenediaminetetraacetic acid and amino acid standards to ensure machine calibration. An in vitro analysis of the samples was done in a water bath using modified methods as described by Tilley and Terry (1963), McDougall (1948), and Mertens (1993). Two donor steers consuming a diet of 50% brome grass hay and 50% wet corn gluten feed (Sweet Bran, Cargill Inc., Blair, NE) provided equal parts rumen fluid for sample inoculation. Between 0.5000 and 0.5040 g of each sample was incubated in 100-mL tubes in triplicate for 48 h. Two incubation runs were conducted for each sample type to account for run-to-run variation (Stalker et al., 2013). Three different corn residues, husk, and husklage samples of known in vivo digestibility values were included as standards for each run. The measured standard values were used to adjust results by averaging the difference between the known and measured digestibility and adding it to the measured sample values. Incubated samples were filtered and dried to obtain in vitro dry matter digestibility (IVDMD) and then filters were incinerated in a 600-°C muffle furnace for 6 h to obtain in vitro organic matter digestibility (IVOMD).

The digestible OM (DOM) of the bales was calculated by multiplying the IVOMD of each bale by the OM content. Estimated DOM intake was calculated similarly, with the IVOMD percentage of each residue waste and refusal sample multiplied by the respective OM content and, then, subtracted from the offered DOM in the bale. The difference between the offered DOM and the remaining DOM in both the waste and refusals represents the DOM which disappeared and was used as an estimate of DOM intake.

### Statistical Analyses

All data were analyzed using SAS 9.2 software for Windows (SAS Institute, Inc., Cary, NC) using the GLIMMIX procedure. Data were first tested for outliers using Cook’s *D* test and one observation was removed from the data set as an outlier. Since pen was the experimental unit for the Latin Square, both animal block (*n* = 2; light and heavy) and period (*n* = 6) were included in the model as fixed effects. Harvest method, chemical treatment, and the interaction between the two factors were also analyzed as fixed effects, and the interaction was removed from the model if *P >* 0.10. Results with a *P* value of <0.05 are considered to be significant, with a tendency to be significant when *P* > 0.05 and < 0.10.

## RESULTS

### Plant Part Composition of Bales

When evaluating the DM contribution of the various corn plant parts to the bales, there was a significant *(P* < 0.01) harvest method by plant part interaction (Table 1). The largest difference in composition of the bales appeared to be the in the amount of cobs. The contribution of cob to the bale was greatest (*P* < 0.01) for 2ROW. The amount of cob was intermediate for EZB, being less (*P* < 0.01) than 2ROW but greater (*P* < 0.01) than CONV. Cob contributed four times more DM in 2ROW bales and twice as much DM in EZB bales compared with CONV bales. The contribution of stem to the bale DM was also greatly impacted by harvest method. Stem proportion was 1.8 times lesser (*P* = 0.03) in 2ROW than EZB and CONV, which did not differ (*P* = 0.28). Leaf content of 2ROW and EZB did not differ (*P* = 0.86) but was lesser (*P* ≤ 0.01) than CONV. The proportion of husk tended (*P* = 0.06) greater for EZB than CONV and did not differ (*P* = 0.73) from 2ROW, while 2ROW and CONV also did not differ (*P* = 0.12). The chaff (unsortable material) was not different (*P* ≥ 0.12) between harvest methods.

**Table 1.** The effect of harvest method (HM)* on the plant part proportion (% of DM) of corn residue bales

	Harvest method				*P* value
Part†	CONV	2ROW	EZB	SEM	HM × Part
Cob	9.1^c^	30.5^a^	18.6^b^	1.99	<0.01
Husk	11.5^a^	15.9^a^	16.9^a^		
Leaf	39.4^a^	31.4^b^	31.9^b^		
Stem	33.3^a^	18.1^b^	30.3^a^		
Chaff	6.7^a^	4.1^a^	2.3^a^		

*Corn residue harvest method is either conventionally harvested rake and bale (CONV), New Holland Cornrower header with two rows of corn plant added to the windrow (2ROW), or the spreader disengaged on the back of the combine (EZBale; EZB).

†Plant parts were hand-sorted according to visual assessment, with leaf sheath included in the leaf portion of the sample. Chaff was also sorted and considered to be material that was sifted through a 1-mm wire mesh screen.

^abc^Means within plant part lacking common superscript differ (*P* ≤ 0.05).

### Residue Quantification

No interaction between harvest method and chemical treatment (*P* = 0.88) was observed for the initial bale weight. There was a difference (*P* < 0.01; SEM ± 21.9) in total bale weight between harvest methods. The 2ROW bales (542 kg DM) were the heaviest (*P* ≤ 0.01), while EZB (506 kg DM) was lighter than 2ROW but greater (*P* ≤ 0.02) than CONV (447 kg DM). Despite the differences in bale weight, when calculated for each pen on a percentage of BW, there was no difference (*P* = 0.89; SEM ± 0.131) in initial offered DM between harvest methods at 1.84%, 1.76%, and 1.80% of BW for 2ROW, EZB, and CONV, respectively. Chemical treatment did not affect (*P* > 0.80) bale weight or initial offered DM on a percentage of BW basis.

There were no interactions (*P* > 0.32) between harvest method and chemical treatment when measuring the wasted and refused residue (Table 2). There was a tendency (*P* = 0.06) for harvest method to affect the amount of wasted residue, with cows fed CONV tending (*P* = 0.08) to waste more residue than those fed 2ROW but not differing (*P* = 0.50) from EZB. Cows fed 2ROW wasted less (*P* = 0.02) than those consuming EZB. Ammoniation reduced (*P =* 0.01) waste by 25% (5.7 percentage units). The amount of refused residue did not differ (*P* = 0.11) by harvest method and chemical treatment did not affect (*P* = 0.26) the amount of refused residue. There was no interaction (*P* = 0.21) between harvest method or chemical treatment for residue disappearance, and both harvest method (*P* = 0.05) and chemical treatment (*P* = 0.03) affected disappearance. Disappearance of CONV was less (*P* = 0.02) than 2ROW but did not differ (*P* = 0.34) from EZB. The disappearance of 2ROW and EZB did not (*P* = 0.12) differ. However, ammoniation increased residue disappearance by 16% (9.5 percentage unit).

**Table 2.** Effect of harvest method (HM) and chemical treatment (CT) on the percentage of corn residue offered that was wasted, refused, or disappeared when fed as whole bales to cows and heifers in a round bale feeder

	Harvest method*			Chemical treatment†			*P* values‡		
	CONV	2ROW	EZB	UNAM	AM	SEM	HM	CT	HM × CT
Percentage of offered residue DM									
Wasted, %	20.9	16.4	22.5	22.8	17.1	2.67	0.06	0.01	0.46
Refused, %	21.4	12.9	14.9	18.3	14.5	4.49	0.11	0.26	0.32
Disappearance**, %	57.7^b^	70.7^a^	62.5^ab^	58.9	68.4	5.52	0.05	0.03	0.21

*CONV: conventionally harvested rake and bale, 2ROW: New Holland Cornrower header with two rows of corn plant added to the windrow, EZB: spreader disengaged on the back of the combine.

†UNAM: nonammoniated corn residue bales; AM: ammoniated residue at 3.7% of DM.

‡Means that share a common superscript are not significantly different from each other (*P* > 0.05).

**Residue disappearance was estimated by subtracting the wasted and the refused residue from the amount of initial offered DM.

### Residue Nutrient Characterization

There were no interactions (*P* > 0.37) between harvest method and chemical treatment for the nutrient content of the residue offered (Table 3). Harvest method did not affect (*P* > 0.58) the DM or CP content of the bales. However, there was an effect (*P* ≤ 0.01) of harvest method on the OM, NDF, ADF, IVOMD, and DOM of the bales. The CONV bales had less (*P* < 0.01) OM, NDF, IVOMD, and DOM and greater ADF content compared with 2ROW and EZB bales, which did not differ (*P* ≥ 0.32). The DM content of the bales did not differ (*P* = 0.37) due to chemical treatment. However, ammoniation had a tendency (*P* = 0.08) to result in a small increase in OM compared with nonammoniated bales. Ammoniation decreased (*P* < 0.01) NDF and increased (*P* < 0.01) CP, IVOMD, and DOM content of the bales but ADF was not affected (*P* = 0.66) by ammoniation.

**Table 3.** Effect of harvest method (HM) and chemical treatment (CT) on nutrient composition of baled corn residue offered, wasted, and refused when fed to cows and heifers as whole bales in a round bale feeder

		Harvest method*			Chemical treatment†			*P* values‡		
		CONV	2ROW	EZB	UNAM	AM	SEM	HM	CT	HM × CT
DM, %	Offered	83.5	83.0	83.7	83.9	82.9	1.10	0.90	0.47	0.90
	Wasted	58.5	59.6	57.7	59.4	57.8	6.4	0.97	0.83	0.41
	Refused	61.3	60.6	56.6	60.6	58.5	6.4	0.87	0.78	0.98
% of DM										
OM, %	Offered	88.1^b^	91.9^a^	92.5^a^	90.1	91.5	0.64	**<0.01**	0.08	0.79
	Wasted	84.0	81.5	81.0	82.5	81.9	1.54	0.34	0.75	0.88
	Refused	84.1	85.0	85.9	83.8	86.2	3.49	0.94	0.56	0.56
NDF, %	Offered	78.9^b^	81.0^a^	81.9^a^	83.7	77.5	0.55	**0.01**	**<0.01**	0.66
	Wasted	77.5	76.3	76.1	77.8	75.4	1.71	0.82	0.24	0.59
	Refused	80.0	80.1	79.7	80.8	79.5	2.62	0.95	0.66	0.12
ADF, %	Offered	57.7^a^	54.6^b^	54.9^b^	55.6	55.8	0.46	**<0.01**	0.66	0.37
	Wasted	54.1	53.1	53.3	52.6	54.3	0.98	0.75	0.15	0.95
	Refused	55.7	54.7	56.4	54.3	56.9	1.73	0.78	0.20	0.24
CP, %	Offered	8.3	8.2	8.2	5.6	10.8	0.10	0.58	**<0.01**	0.99
	Wasted	7.3	7.5	7.6	6.5	8.4	0.63	0.94	**0.02**	0.30
	Refused	7.1	7.3	7.6	6.6	8.1	0.69	0.90	0.08	0.16
IVOMD, %	Offered	50.0^b^	54.6^a^	54.4^a^	46.9	59.1	0.65	**<0.01**	**<0.01**	0.76
	Wasted	41.9	42.5	41.6	41.5	42.6	1.49	0.91	0.51	0.80
	Refused	39.3	41.2	42.7	40.7	41.4	1.91	0.47	0.76	0.90
DOM, %	Offered	44.1^b^	50.2^a^	50.4^a^	42.3	54.1	0.67	**<0.01**	**<0.01**	0.52
	Wasted	35.2	34.7	33.9	34.3	35.0	1.52	0.83	0.68	0.79
	Refused	33.2	35.2	36.7	34.3	35.8	2.33	0.58	0.60	0.80

*CONV: conventionally harvested rake and bale, 2ROW: New Holland Cornrower header with two rows of corn plant added to the windrow, EZB: spreader disengaged on the back of the combine (EZBale).

†UNAM: nonammoniated corn residue bales; AM: ammoniated residue at 3.7% of DM.

‡Means that share a common superscript are not significantly different from each other (*P* > 0.05).

No interactions between harvest method and chemical treatment were noted (*P* ≥ 0.12) in the nutrient content of either waste or refusals. No effect of harvest method (*P* ≥ 0.34) was observed on any of the nutrients measured for both wasted and refused material (Table 3). Chemical treatment did not affect (*P* ≥ 0.15) the nutrient content (DM, OM, NDF, ADF, IVOMD, and DOM) of waste or refusals with the exception of CP content. The CP content of waste from ammoniated bales was greater (*P* = 0.02) than nonammoniated bale waste. Similarly, the CP content of the refusals from the ammoniated bales tended (*P* = 0.08) to be greater than refusals from the nonammoniated bales.

Based on the difference between what was offered and what remained in the waste and refusals, the estimated daily nutrient intake was calculated (Table 4). When calculated as a percentage of average pen BW, there was a tendency (*P* = 0.06) for an interaction between harvest method and chemical treatment for estimated daily DM intake (DMI). When not ammoniated, DMI of CONV was less than 2ROW and EZB, which did not differ (*P* = 0.87). Ammoniation resulted in DMI of 2ROW to be greater (*P* < 0.01) than all other treatments. Although numerical increases in DMI due to ammoniation were noted, CONV and EZB intake did not appear to respond to ammoniation statistically, with no difference in (*P* ≥ 0.16) intake of the nonammoniated and ammoniated residue within harvest method. When DMI was evaluated on a kilogram per cow per day basis, the interaction between harvest method and chemical treatment was not significant (*P* = 0.11), although similar numerical trends to DMI on a BW basis were observed.

**Table 4.** Effect of harvest method (HM) and chemical treatment (CT) on estimated nutrient consumption by cows fed whole bales of corn residue in a round bale feeder

	Unammoniated			Ammoniated†				*P* values		
	CONV*	2ROW	EZB	CONV	2ROW	EZB	SEM	HM	CT	HM × CT
DMI, %BW	0.94^y^	1.27^x^	1.29^x^	1.15^xy^	1.81^w^	1.31^x^	0.117	<0.01	<0.01	0.06
Kilogram per cow per day										
DM	5.03	7.22	6.98	6.44	10.11	7.33	0.64	<0.01	<0.01	0.11
OM	4.57^d^	6.79^bc^	6.77^bc^	5.72^cd^	9.58^a^	7.25^b^	0.52	<0.01	<0.01	0.05
NDF	4.24^z^	6.11^x^	6.28^x^	4.79^yz^	7.98^w^	5.77^xy^	0.51	<0.01	0.10	0.06
ADF	3.12^x^	3.92^x^	3.89^x^	3.72^x^	5.65^w^	3.99^x^	0.39	<0.01	0.01	0.09
CP	0.25	0.41	0.33	0.79	1.10	0.91	0.053	<0.01	<0.01	0.27
DOM	2.17^d^	3.62^c^	3.48^c^	3.56^c^	6.04^a^	4.99^b^	0.22	<0.01	<0.01	0.03

*CONV: conventionally harvested rake and bale, 2ROW: New Holland Cornrower header with two rows of corn plant added to the windrow, EZB: spreader disengaged on the back of the combine (EZBale).

†Corn residue ammoniated at 3.7% of DM.

^abcd^Means that share a common superscript are not significantly different from each other (*P* > 0.05).

^wxyz^Means that share a common superscript tend to not differ from each other (*P* > 0.10).

There was an interaction (*P* ≤ 0.05) between harvest method and chemical treatment for OM and DOM intake, and there tended (*P* ≤ 0.09) to be interactions for NDF and ADF intake when evaluated on a kilogram per cow per day basis. In general, these responses followed the same pattern as DMI on a BW basis. For OM and NDF, when not ammoniated, intake of CONV was less (*P* ≤ 0.01) than EZB, while EZB and 2ROW did not differ (*P* ≥ 0.80). Ammoniation tended (*P* = 0.08) to increase OM intake of CONV, but not (*P* = 0.39) NDF intake, and did not increase OM or NDF intake of EZB (*P* ≥ 0.44). Ammoniation resulted in a significant increase (*P* < 0.01) of both OM and NDF intake for 2ROW. Thus, OM and NDF intake of ammoniated 2ROW was greater (*P* ≤ 0.01) than all other treatments. The three harvest methods did not differ (*P* ≥ 0.13) in ADF intake when not ammoniated, and ammoniation did not increase (*P* ≥ 0.23) the intake of CONV or EZB. Similar to other nutrients, ammoniation did increase (*P* < 0.01) the ADF intake of 2ROW residue, resulting in ammoniated 2ROW being greater (*P* < 0.01) than all other treatments.

The intake of DOM was similar to most of the other nutrients when not ammoniated, where CONV was less (*P* < 0.01) than 2ROW and EZB, which did not differ (*P* = 0.61). However, DOM intake of all three harvest methods appeared to have an increase (*P* < 0.01) in intake due to ammoniation, and this response appeared to be greatest (*P* < 0.01) for 2ROW residue.

Unlike the others nutrients, there was no interaction (*P* = 0.26) for CP intake. Harvest method did significantly (*P* < 0.01) affect CP intake. The CP intake of CONV was less (*P* < 0.01) than 2ROW and tended (*P* = 0.06) to be less than EZB, with 2ROW being greater (*P* = 0.02) than EZB. Ammoniation increased (*P* < 0.01) CP intake for all harvest methods.

## DISCUSSION

While harvest method resulted in the nutrient content of the corn residue offered in round bale feeders to differ due to variation in the proportion of plant parts, it is interesting to note that the nutrient content of the waste and refusals appeared to be similar among harvest methods. Also, with the exception of CP, the nutrient content of the waste and refusals was not affected by chemical treatment. These data suggest that the cows were able to be selective in their intake, consuming the most digestible plant parts in the bale, and that differences in intake among harvest methods appear to be driven by availability of digestible material. The plant part composition may also explain the differences in apparent response to ammoniation. The 2ROW appeared to have the greatest increase in intake due to ammoniation and had a lower amount of stem than both CONV and EZB. However, little work has been conducted to evaluate the response of different corn plant parts to ammoniation.

In the current study, it may be appropriate to consider waste (material that falls outside of the feeder) and refusals (material remaining in the feeder) together as overall “uneaten material” for corn residue as the unpalatability of certain corn plant parts (i.e., stem) will make complete consumption of the bale unlikely. In order to compare the cost of feeding corn residue to that of feeding hay, the difference in “uneaten material” should be taken into account.

When cattle were fed tall fescue (7.5% CP; 36% ADF) in round feeders with bottom paneling similar to the feeders in the present study, but with tapered sides, they wasted 13.6% and refused another 13.5% of the hay offered, resulting in a total of 27% of the bale remaining uneaten (Moore and Sexten, 2015). Walker et al. (2013) reported waste plus refusals of bermudagrass hay (8% to 9% CP; 38% to 44% ADF) to range from 20% to 30% when fed in ring feeders. In the present study, the amount of uneaten material was 42% for nonammoniated residue and 32% for ammoniated residue. Thus, while the nonammoniated residue has greater losses than feeding hay, the ammoniated residue appears to be similar to what would be expected with feeding lower-quality hay. However, despite the fact that there was not a harvest method by chemical treatment interaction (*P* = 0.21; SEM ± 5.0), there were some large variations among harvest methods in response to ammoniation when evaluating the amount of “uneaten material” (refusals and waste). The amount of uneaten material was 47%, 38%, and 38% for nonammoniated CONV, 2ROW, and EZB, respectively. When ammoniated, the amount of uneaten material was 37%, 20%, and 38% of the offered corn residue for CONV, 2ROW, and EZB, respectively. The reduction in uneaten material for 2ROW when ammoniated would have a large impact on its cost when comparing forages on a consumed basis.

A 590-kg cow (mean BW in this study was 582 kg) will require between 5.0 and 6.2 kg Total digestible Nutrients (TDN) per day to meet her energy needs in mid and late gestation and her CP requirement would be between 0.73 and 0.91 kg/d. Assuming that DOM is equal to TDN, the nonammoniated residue, regardless of harvest method, would not have met the cow’s energy requirements. Likewise, the nonammoniated residues would have failed to meet her protein requirements. Thus, if feeding nonammoniated corn residue, regardless of harvest method used, there would be a need to supplement both energy and protein to pregnant cows. However, it should be noted that, in this study, the selected diet of the cows consuming the nonammoinated residue would not be predicted to meet ruminal degradable protein requirements; thus, providing supplemental degradable protein may result in increased intake and, subsequently, reduce the need for supplemental energy.

However, ammoniation increased both the CP and DOM in the residue bales and the cows were able to select a higher-quality diet than when offered nonammoniated bales. The selective harvest methods, 2ROW and EZB, would meet the energy requirements of mature cows in mid-gestation. While the CP intake of all of the ammoniated residues was enough to meet the CP requirements during mid-gestation, a small amount of energy supplementation would be needed in late gestation even with the ammoniated selectively harvested residue (2ROW and EZB).

## CONCLUSIONS

Ammoniation appeared to have a large impact on reducing waste and refusals and increasing the nutrients consumed. Combining ammoniation and selective harvest methods of corn residue resulted in estimated DOM and CP intakes of cows to be sufficient to meet the energy and protein requirements of a mature cow in mid-gestation, but additional supplementation to meet energy requirements would be needed in late gestation. With the exception of corn residue harvested using a method that reduces the amount of stem (2ROW) that was ammoniated, the unconsumed material of round bales offered in a ring feeder is likely to be greater than hay, and this difference should be accounted for when comparing the cost of corn residue fed in a bale feed relative to the cost of hay.
